# A systematic review of the neurobiological effects of theta-burst stimulation (TBS) as measured using functional magnetic resonance imaging (fMRI)

**DOI:** 10.1007/s00429-023-02634-x

**Published:** 2023-04-19

**Authors:** Melissa Kirkovski, Peter H. Donaldson, Michael Do, Bridgette E. Speranza, Natalia Albein-Urios, Lindsay M. Oberman, Peter G. Enticott

**Affiliations:** 1 Institute for Health and Sport, Victoria University, Melbourne, VIC Australia; 2 Cognitive Neuroscience Unit, School of Psychology, Deakin University, Geelong, VIC Australia; 3 National Institute of Mental Health, National Institutes of Health, Bethesda, MD USA

**Keywords:** Transcranial magnetic stimulation (TMS), Repetitive transcranial magnetic stimulation (rTMS), Theta burst stimulation (TBS), Functional magnetic resonance imaging (fMRI), Non-invasive brain stimulation (NIBS), Inter-individual variability

## Abstract

Theta burst stimulation (TBS) is associated with the modulation of a range of clinical, cognitive, and behavioural outcomes, but specific neurobiological effects remain somewhat unclear. This systematic literature review investigated resting-state and task-based functional magnetic resonance imaging (fMRI) outcomes post-TBS in healthy human adults. Fifty studies that applied either continuous—or intermittent—(c/i) TBS, and adopted a pretest–posttest or sham-controlled design, were included. For resting-state outcomes following stimulation applied to motor, temporal, parietal, occipital, or cerebellar regions, functional connectivity generally decreased in response to cTBS and increased in response to iTBS, though there were some exceptions to this pattern of response. These findings are mostly consistent with the assumed long-term depression (LTD)/long-term potentiation (LTP)-like plasticity effects of cTBS and iTBS, respectively. Task-related outcomes following TBS were more variable. TBS applied to the prefrontal cortex, irrespective of task or state, also produced more variable responses, with no consistent patterns emerging. Individual participant and methodological factors are likely to contribute to the variability in responses to TBS. Future studies assessing the effects of TBS via fMRI must account for factors known to affect the TBS outcomes, both at the level of individual participants and of research methodology.

## Introduction

Transcranial magnetic stimulation (TMS) protocols rely on a time-varying magnetic field to produce an electrical current that non-invasively depolarizes axons underlying a specialized coil held against the scalp (Barker et al. 1985; Klomjai et al. 2015). This technique has been used since 1985 to transiently probe cortical excitability in human participants (Barker et al. 1985). Repetitive (r)TMS protocols, in which multiple TMS pulses are delivered in succession, have longer-lasting neuro-modulatory effects, and thereby have broader applications for basic neuroscience and also significant clinical applications (Klomjai et al. 2015). Theta burst stimulation (TBS) is a rTMS protocol that was first developed for human application by Huang and colleagues (2005). Unlike conventional rTMS protocols, in which stimuli (i.e., pulses) are spaced identically with the inter-stimulus-interval ranging between 1 and 25 Hz (Klomjai et al. 2015), pulses administered during TBS are patterned based on the brain’s natural hippocampal theta rhythm (Klomjai et al. 2015; Suppa et al. 2016). This protocol is suggested to modulate gamma oscillations, commonly referred to in the literature as theta-gamma coupling (Cárdenas-Morales et al. 2010).

The standard TBS paradigm involves administering TMS in three-pulse 50 Hz “bursts” of stimulation repeated every 200 ms (5 Hz, “theta” burst frequency), mimicking the rhythm of theta-gamma coupling (Huang et al. 2005). Continuous TBS (cTBS) involves uninterrupted TBS delivery, typically across 40 s (600 pulses). When administered over the primary motor cortex (M1), the effects of cTBS are generally considered to suppress cortico-spinal activity, as measured via motor evoked potential (MEP) amplitude (Huang et al. 2005; Chung et al. 2016). In contrast, for intermittent TBS (iTBS), whereby 8 s inter-train intervals follow 2 s trains of stimulation, typically for 190 s (600 pulses), a facilitatory effect on MEPs is described (Chung et al. 2016; Huang et al. 2005).

Although the neurological mechanisms underpinning cTBS and iTBS are not fully characterized (Di Lazzaro et al. 2005, 2008), TBS neuromodulation is likely underpinned by long-term depression (LTD)—and long-term potentiation (LTP)—like mechanisms (Huang et al. 2011). Studies have shown that TBS effects originate in the cortex (Di Lazzaro et al. 2005, 2008) and are *N*-methyl-d-aspartate receptor dependent (Huang et al. 2007). Though both TBS protocols are modelled on theta-gamma coupling, the variations in protocol have differential effects on the TMS-induced corticospinal volleys, and affect different populations of neurons. First, direct stimulation of the pyramidal tract results in a corticospinal volley labelled the D-wave. Following this, synaptic activation induces indirect (I)-waves. It is the I-wave(s) which appear to be affected by variation in the TBS protocol. cTBS preferentially suppresses the first (I1) component of the I-wave, eliciting an overall suppression, or LTD-like effect (Di Lazzaro et al. 2005), while later components of the I-wave and also the D-wave appear unaffected. iTBS, in contrast, increases later I-wave amplitude, producing an LTP-like effect (Di Lazzaro et al. 2008). The exact neurobiological mechanisms which underpin these responses to variation in TBS protocol remain elusive; however, several comprehensive reviews provide more detailed overviews on the mechanistic effects of TBS (Cárdenas-Morales et al. 2010; Klomjai et al. 2015; Suppa et al. 2016).

Notably, while the seminal work by Huang et al. (2005) described facilitatory and inhibitory effects of iTBS and cTBS, respectively, many consecutive studies emphasize a high degree of inter-individual variability in the behavioral and neurobiological response to TBS paradigms (Do et al. 2018; Chung et al. 2016; Hamada et al. 2013; Corp et al. 2020; Jannati et al. 2017; López-Alonso et al. 2014), and while the factors surrounding this observed variability remain largely unknown (Ridding and Ziemann 2010), important work is being conducted to elucidate this (Corp et al. 2020).

While it was originally considered that TMS delivered at such a high-frequency as TBS might yield superior outcomes to conventional rTMS protocols (Huang et al. 2005; Suppa et al. 2016), TBS is increasingly being used in clinical and non-clinical research settings as it is delivered faster and at lower intensities than conventional rTMS (Huang et al. 2005; Chung et al. 2015, 2016) while yielding equivalent, if not enhanced, neuroplastic effects (Chung et al. 2015). The safety and tolerability profile of TBS in both adult and paediatric samples is now well-established (Oberman et al. 2011; Hong et al. 2015; Rossi et al. 2009, 2021). This patterned rTMS protocol is commonly applied to probe brain-behaviour relationships (Demeter 2016), is considered a viable alternative to conventional rTMS as a biomedical intervention for major depressive disorder (Chung et al. 2015; Bulteau et al. 2022; Blumberger et al. 2018), and has been trialled for anxiety-related disorders, psychotic symptoms, and dependence disorders (see Rachid 2017 for a review). From a clinical perspective, the shorter duration of TBS protocols allow for many more sessions/treatments to be delivered daily within clinics, allowing greater access for patients (Chung et al. 2015), and also increases the feasibility of accelerated treatment protocols (Cole et al. 2020; Xiao et al. 2019; Sonmez et al. 2019).

Much of what is known regarding the neurobiological and mechanistic effects of TBS, like TMS more broadly, is derived from research investigating the motor cortex. This is primarily due to the relative accessibility of measurable outcomes, such as MEPs recorded via electromyography in peripheral muscles (Chung et al. 2016; Di Lazzaro et al. 2005, 2008; Huang et al. 2005). As with all TMS protocols, the extent to which this knowledge translates to regions beyond the motor cortex is unclear. Despite this, the past decade has seen rapid growth in research implementing TBS protocols outside of the motor cortex, both experimentally and clinically. It is well established that the cytoarchitecture of different brain regions, however, varies widely, which consequently affects signal transmission (van den Heuvel et al. 2015), and is, therefore, likely to have implications for the response to TBS.

Functional magnetic resonance imaging ([f]MRI) can be immensely beneficial in elucidating the neurobiological effects of TBS. Such protocols can provide indications of TBS-induced alterations in regional excitability and network connectivity/reactivity, beyond the motor cortex, and with good spatial resolution. In this review, we sought to describe and synthesise the literature investigating the neurobiological after-effects of a single session of TBS in non-clinical adult populations, as measured via fMRI.

## Methods

The review protocol was registered with PROSPERO (PROSPERO 2020 CRD42020150589) and was conducted in alignment with the Preferred Reporting Items for Systematic Reviews and Meta-Analysis (PRISMA) guidelines (Moher et al. 2009, 2015).

### Search strategy

We searched for research papers (peer-reviewed [published or in press], pre-print, or thesis) published in English, and did not impose limits on year of publication. Scopus, Ovid Medline, and Google Scholar were initially searched on 16th September 2019 using the following search terms: (“theta burst stimulation” or “TBS” or “continuous theta burst stimulation” or “cTBS” or “intermittent theta burst stimulation” or “iTBS”) and (“magnetic resonance imaging” or “MRI” or “functional magnetic resonance imaging” or “fMRI” or “functional MRI” or “magnetic resonance spectroscopy” or “MRS” or “neuroimaging”). One reviewer (MK) completed and compiled searches into the Rayyan database (Ouzzani et al. 2016). A final iteration of the searches was completed and updated on 10th October, 2022.

### Eligibility criteria

Research was considered eligible for review if either cTBS or iTBS was applied to any part of the human cortex, with the MRI outcomes listed above recorded following stimulation. Studies were required to have adopted a pretest–posttest or sham-controlled design.

Only outcomes for neurotypical (i.e., non-clinical) adults, aged 18 and above, were reviewed. Research was not excluded if clinical (i.e., neuropsychiatric, neurological, or neurodevelopmental disorders) or paediatric comparisons were also presented; however, this information is not reported on in the present review. This decision was made to avoid confounds associated with the presence of neuropathophysiology and/or neurodevelopmental factors.

### Screening

Title and abstract screening were completed by two of the authors (MK and PHD) via Rayyan (Ouzzani et al. 2016). The reviewers were blind to each other’s decisions. In instances where the title and abstract did not provide sufficient information to determine eligibility, the full text (methods section) of the manuscript was reviewed. Upon completion of the initial screening, results were unblinded and any discrepancies were resolved via discussion between the two reviewers. Where consensus could not be reached, a third reviewer (PGE) was consulted.

### Risk of bias assessment

Risk of bias was assessed by one researcher (MK) using the Cochrane Risk of Bias assessment tool, Version 2 (RoB 2) (Higgins et al. 2016; Sterne et al. 2019). The use of one risk of bias assessor deviates from the protocol outlined in our Prospero Registration (PROSPERO 2020 CRD42020150589) for this review, where it was proposed that risk of bias assessment would be conducted by two researchers. The decision to make this change was in large part a consequence of resource limitations due to the COVID-19 pandemic. Following completion of this assessment, the final judgements were discussed and agreed upon with the senior author (PGE).

Refer to Table 1 for a summary of this assessment. Information provided within some manuscripts indicates that data came from the same sample/study (manuscripts linked/highlighted in Table 1). In these instances, if information was unclear or not reported in one manuscript, but relevant information could be extracted from another manuscript reporting on the same sample/protocol, this information/assessment was transferred between papers.

**Table 1:**
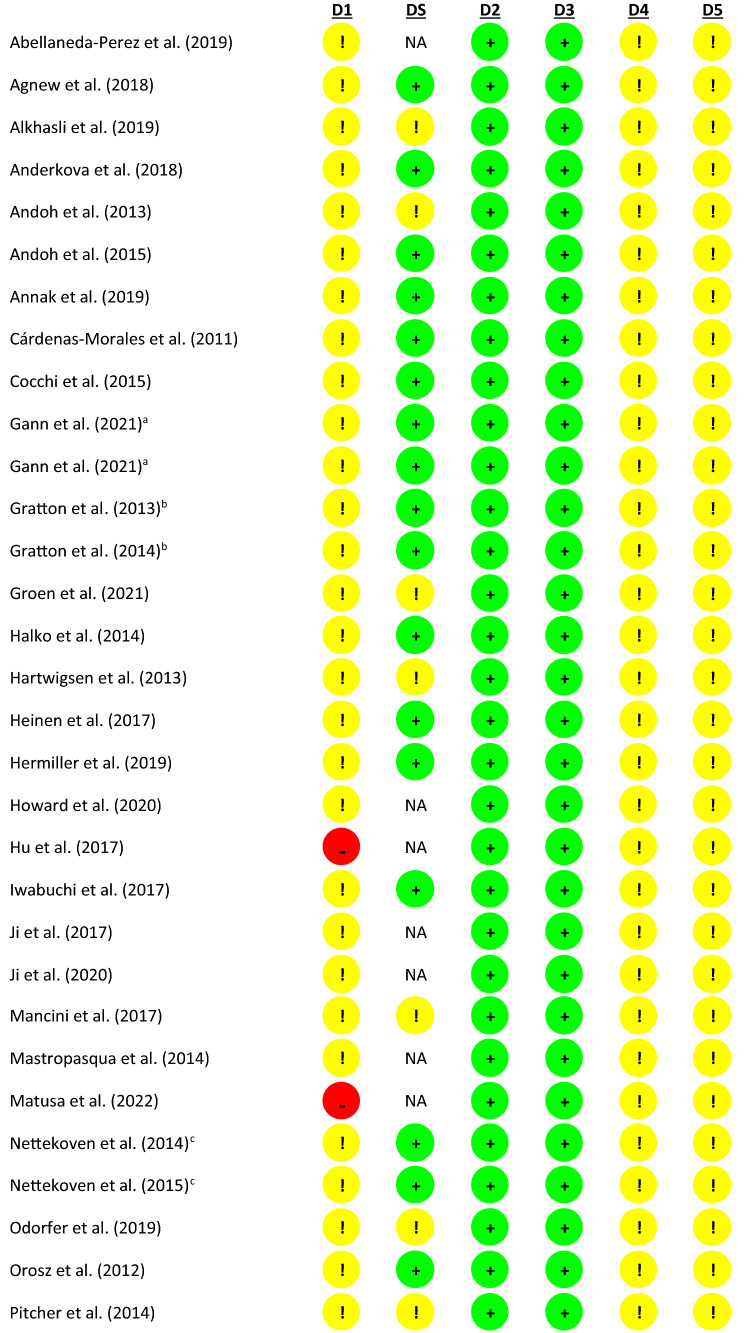

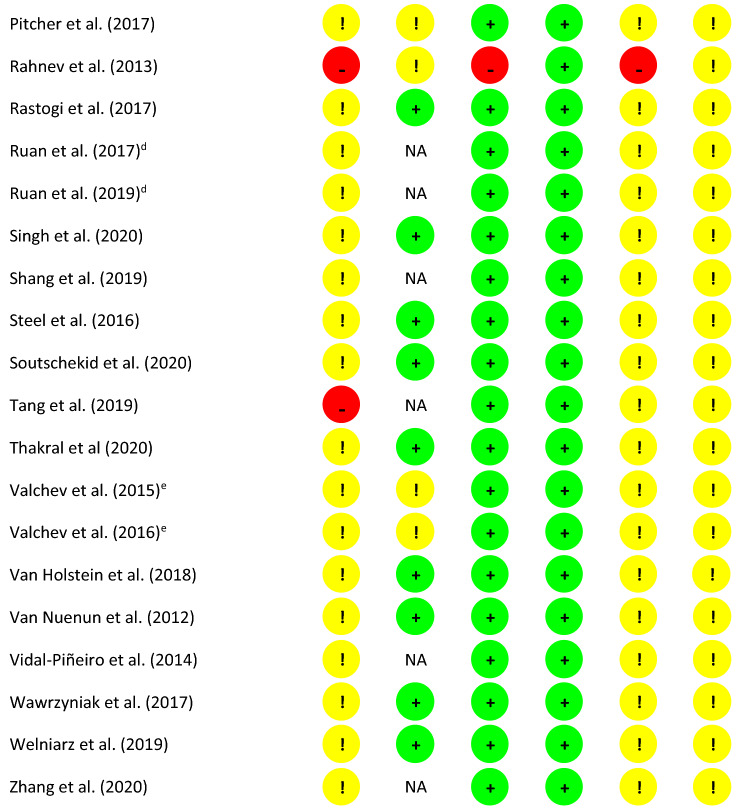
Summary of Risk of Bias Assessment Table, based on Cochrane Risk of Bias assessment tool, Version 2

D1 = Randomisation process, DS = Risk of bias arising from period and carryover effects in a cross-over trial, D2 = Deviations from the intended interventions, D3 = Missing outcome data, D4 = Measurement of the outcome, D5 = Selection of the reported result

^a,b,c,d,e^Information provided within these manuscripts indicates that data come from the same sample/study

The majority of reviewed studies adopted cross-over designs (*n* = 37), rather than parallel designs (*n* = 10), or single-arm designs (*n* = 3). For cross-over studies, the RoB 2 considerations for cross-over trials (https://sites.google.com/site/riskofbiastool/welcome/rob-2-0-tool/rob-2-for-crossover-trials?authuser=0) are also provided. One study (Shang et al. 2019) implemented a cross-over design whereby two groups (active/sham TBS) crossed over into a no-TBS session. For the purpose of this assessment, this study was considered a parallel design.

Regarding *Domain 1: Randomisation Process*, while studies reported that participants were randomly allocated to conditions (where appropriate to study design), only one study provided sufficient information regarding the use of a “simple” randomisation protocol (Abellaneda-Pérez et al. 2019). For studies adopting single-arm designs, the risk of bias in this domain was considered high, given that randomisation was not possible. No studies reported concealment efforts or processes. As mentioned above, for studies reporting cross-over designs we also considered *Domain S: Bias Arising from Period and Carryover Effects* of the RoB 2. Regarding period effects, while only one study (Valchev et al. 2016) reported precise information regarding sequence allocation, all others indicated counterbalancing of sessions. Therefore, it is probable that the number of participants allocated to each sequence was equal or nearly equal. The washout period between sessions, and therefore, the potential for carryover effects, varied across studies. At a minimum, the reviewed studies report spacing sessions *at least one day apart*, while others implemented a week washout period to further reduce the risk of carryover effects. In healthy (neurotypical) individuals, the effects of TBS are generally considered to last up to approximately one hour (Wischnewski and Schutter 2015). While there is evidence from conventional rTMS studies to show that cumulative metaplastic effects might exist when sessions are spaced 24 h apart (Bäumer et al. 2003; Maeda et al. 2000), another study reports that when cTBS is applied 24 h apart, this cumulative metaplastic effect was not significant among non-clinical controls (Oberman et al. 2016). We therefore considered a minimum of 24 h to be an appropriate washout period for the studies reviewed here. A number of the reviewed studies reported that sessions were conducted on different days, but do not provide an indication of the time between sessions. It cannot, therefore, be ruled out that sessions might have occurred less than 24 h apart (i.e., late afternoon session followed by a morning session). In these instances, not enough information had been provided to make a clear judgement regarding risk.

When blinding was included in study protocols, the details around such procedures were, in many instances, insufficient. For example, for “double-blind” designs, without further detail, it was unclear which researchers (i.e., those administering TBS, those collecting outcome measures, or those analysing data) were blinded to condition. Only one study (Orosz et al. 2012) specified blinding of the researcher obtaining the scans. Two others reported researcher blinding during the administration of clinical assessments (Odorfer 2019; Singh et al. 2020), outcomes of which were not considered in this review. Despite this, none of the reviewed studies reported *Deviations from the Intended Intervention,* and analyses appropriately considered group assignment, so the risk of bias in this regard *(Domain 2)* was still regarded as low. Risk of bias was also considered low across all studies regarding *Missing Outcome Data (Domain 3)* as there was no indication of condition-specific attrition across any of the studies. On *Domain 4: Measurement of the Outcome,* the tool’s algorithm pointed towards a low risk of bias across all studies as, despite lack of researcher blinding, we do not report on any researcher guided outcomes in this review, and the outcomes (imaging protocols) were identical across conditions. Therefore, it might be considered unlikely that “assessment of the outcome [would] have been influenced by knowledge of intervention received.” For most studies, however, the researchers were not blind to condition. As a result of this, there exists a possibility that their interactions with participants could have been subtly different between conditions, alerting participants to conditions or expectation. Finally, there were some concerns for all studies across *Domain 5: Selection of Results*. None of the studies specifically report having performed blind analysis. While all results appear to be in line with the reported analysis plan, this lack of blinding poses some risk of selective reporting of analyses or results.

Our assessment of the risk of bias in the reviewed studies highlighted several areas of unclear reporting in the literature, making the risk of bias assessment difficult. Clearer reporting across many domains is imperative going forward. Further, where possible, we strongly encourage blinding of researchers, during assessment and analysis, and clearer reporting of these practices.

### Analysis

A systematic/narrative approach was adopted for this review, as there is a pressing need for a clear evaluation and summary of the relevant literature in the field. This consolidation of the available literature will provide brain stimulation researchers with much-needed direction when planning future TBS studies that involve a neuroimaging component. It was decided that a meta-analytic approach would not be appropriate for this review given the multiple sources of heterogeneity associated with these studies in terms of design, stimulation site, and outcomes measures.

## Results

**Fig. 1:**
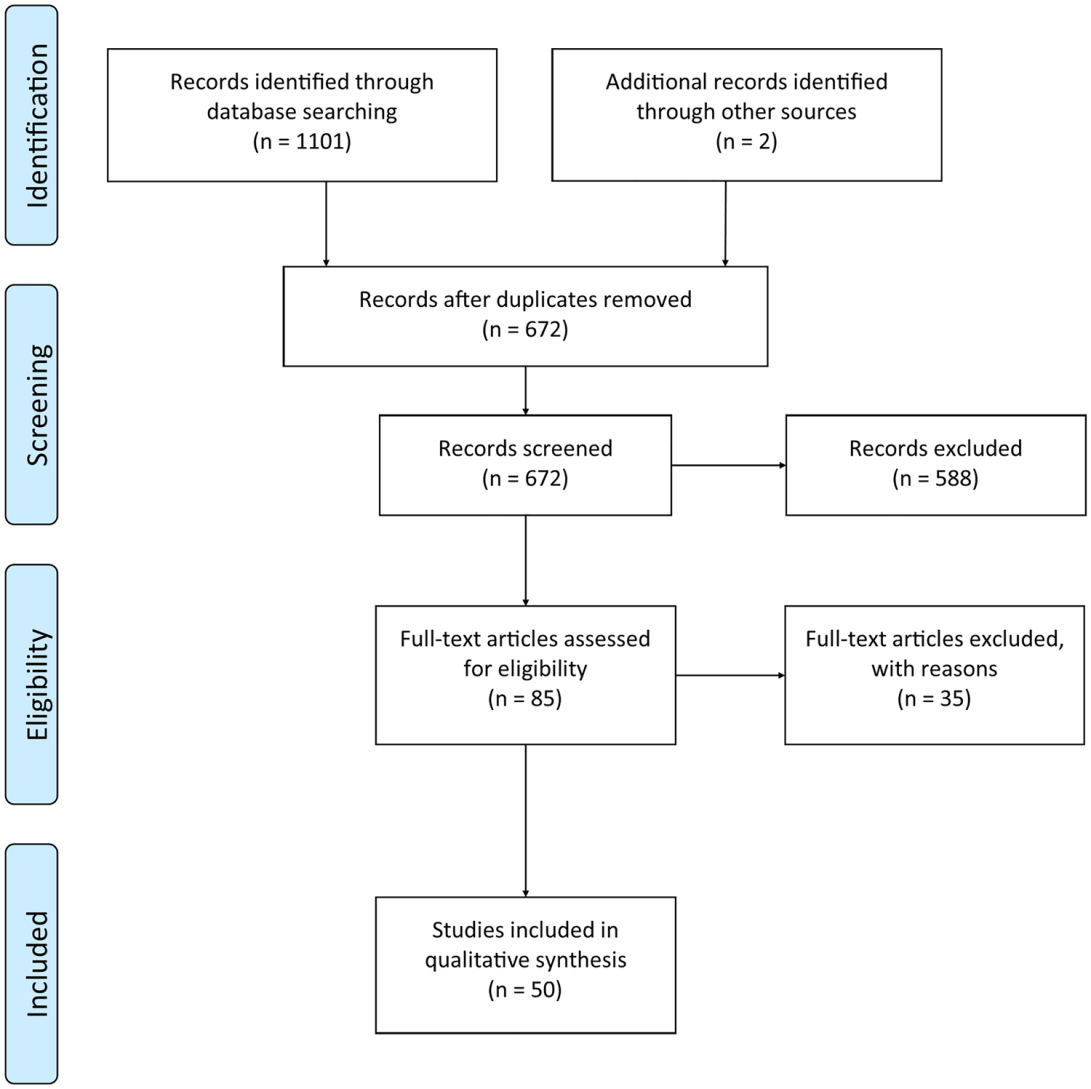
PRISMA flow diagram for search, screening, and manuscript selection. Adapted from Moher et al. (Moher et al. 2009)

Our searches initially identified a total of 1101 manuscripts. One additional manuscript (Gratton et al. 2014) was identified via the references presented within the reviewed manuscripts, and one was referred to us (Singh et al. 2020). 672 remained following the removal of duplicates, and these were then screened according to the criteria described above, resulting in 85 manuscripts that were full text screened for eligibility. Of these, 35 were excluded for the following reasons: not meeting eligibility criteria (*n* = 27), full text published in a language other than English (*n* = 1), and duplication of results (i.e., peer-reviewed manuscript from within a thesis [*n* = 4 theses excluded]). One manuscript (Zhang et al. 2019) was also excluded as the protocol applied deviated from the purpose of this review by applying cTBS immediately followed by iTBS. Additionally, two manuscripts employed magnetic resonance spectroscopy (MRS) as outcome measures, and one reported both MRS and fMRI outcomes. Therefore, at this point, it was decided that MRS outcomes (*n *= 2 manuscripts) would be excluded from this review as the limited research in this area would limit our ability to make any informed interpretations about the effects of TBS on MRS outcomes. This resulted in a total of 50 manuscripts being included in this review. Results of the search and screening process are presented in Fig. 1. Relevant sample, protocol details, and a general indication of the results from included studies are summarised in Table 2.

**Table 2:** Summary of reviewed studies and indication of results

Reference	Sample size, sex ratio (M:F), age (years)	Study design	TBS protocol, parameters^a^, Stimulation Intensity	Method for locating target site [active control]	*Outcome Protocol* [Time to return to scanner] *Paradigm* Summary of results
*TBS to the motor cortex*					
Agnew et al. (2018)	*N* = 16 23–49 years ^b^	Pretest–posttest, within-subjects	cTBS; Total pulses: 300 40% MSO	Neuronavigation (anatomical/coordinate) – (R) ventral premotor cortex (MNI = 54, − 2, 44) – [vertex]: middle of nasion and inion	*Task (auditory emotion)-based fMRI* **[fMRI 5 min post cTBS]** ***BOLD*** No effects at target site or contralateral homologue ↑ at a (R) post- and pre-central gyri ↑ inferior frontal gyrus (pars opercularis and triangularis), (L) supplementary motor area, cerebellar vermis, (R) parahippocampal gyrus, bilateral inferior parietal lobe (angular gyrus), bilateral superior and right middle frontal gyri and (R) postcentral gyrus ↓ (L) hippocampus, (R) middle cingulate cortex, (R) precuneus, (L) supramarginal gyrus, (R) supplementary motor area, (R) inferior frontal gyrus (pars triangularis) and (R) rolandic operculum
Annak et al. (2019)	*N* = 16 (6:10) 23.8 ± 2.3	Pretest–posttest, within-subjects, sham-controlled	cTBS 80% AMT	Neuronavigation (functional activation): – (L) primary motor cortex; FDI representation – (L) secondary somatosensory cortex	*Task (nociceptive stimulation; chemosensory pain model)-based fMRI* **[time to return to scanner unclear/not reported]** ***BOLD*** (L) primary motor cortex: ↓ BOLD at rolandic operculum, insula and postcentral gyrus Strength of relationship between BOLD signal and stimulus strength reduced post cTBS to (L) primary motor cortex No effects of stimulation to (L) secondary somatosensory cortex
Cárdenas-Morales et al. (2011)	*N* = 17 (17:0) 27.3 ± 2.6	Pretest–posttest, within-subjects	iTBS 90% AMT	Hot-spot – (L) primary motor cortex; APB representation	*Task (choice reaction)-based and resting-state fMRI* **[immediate]** ***BOLD and CBF*** Task: ↓ BOLD at (L) primary motor cortex, and (R) primary motor cortex, primary somatosensory cortex, rostral parts of (R) superior and inferior parietal gyrus, and the (R) premotor area No effects at rest
Cocchi et al. (2015)	*N* = 23 (11:12) 23.0 ± 3.0	Pretest–posttest, within-subjects	cTBS iTBS 80% AMT	Hot-spot – (R) primary motor cortex; APB	*Resting-state fMRI* **[5 min]** ***Functional Connectivity*** cTBS: ↓ participation index (PI) and ↑ within module degree (WMD) at motor and somatosensory cortices, ↑ PI and ↓ WMD at insula, striatum, and (L) temporal cortex iTBS: No effects of iTBS on PI or WMD
Hu et al. (2017)	*N* = 36 (17:19) 20–34 years^b^	Pretest–posttest, within-subjects	cTBS 70% RMT	Hot-Spot – (R) primary motor cortex; FDI	*Resting-state fMRI* **[30 min]** **Functional connectivity** Bilaterally ↓ ALFF, fALFF and ReHO along the postcentral gyrus at sites controlling the (L) face and limbs and (L) and (R) trunk
Ji et al. (2017)	*N* = 19 (6:13) 22.7 ± 2.1	Pretest–posttest, within-subjects	cTBS (3 runs, 15 min apart) 70% RMT	Neuronavigation (anatomical/coordinate) – (L) supplementary motor area (MNI = − 6, − 6, 77)	*Resting-state fMRI* **[immediate]** ***BOLD and Functional Connectivity*** No local effects ↓ rsFC at the (L) inferior frontal gyrus, and (L) supplementary motor area/middle cingulate cortex, maintained for 6.1 min in the (L) inferior frontal gyrus and 6.6 min in the (L) supplementary motor area/middle cingulate cortex
Ji et al. (2020)	Primary study: *N* = 33 (17:16) Secondary (validation) study: *N* = (11:5) 20.4 ± 0.51	Pretest–posttest, between-subjects, sham controlled (validation study)	cTBS: 3 runs, 15 min apart 70% RMT	Neuronavigation (anatomical/coordinate) (L) supplementary motor area (MNI = − 6, − 6, 77)	*Resting-state fMRI* **[immediate]** ***Functional Connectivity*** Primary study: ↓ rsFC at the bilateral cerebellum immediately post stimulation (T2). ↑ rsFC at the paracentral gyrus at T3 (immediately post T2) Validation study: ↑ rsFC at the paracentral gyrus at T3 (immediately post T2)
Matusa et al. (2022)	*N* = 25 27–43 years	Pre-test – posttest, within subjects	cTBS 80% AMT	Hot-spot – (L) primary motor cortex; APB	*Resting-state fMRI* **[15 min]** ***Network activity*** No difference in resting state network activity per-post cTBS
Nettekoven et al. (2014)	*N* = 16 (7:9) 27.0 ± 3.0	Pretest–posttest, sham-controlled, within-subjects, repeated measured (time-points)	iTBS: 3 runs, 15 min apart 70% RMT	Hot-spot – (L) primary motor cortex; APB	*Resting-state fMRI* **[~ 3 min]** **Functional connectivity** ↑ rsFC between primary motor cortex and various sensorimotor regions, with local maxima consistently at bilateral supplementary motor area and dorsal premotor cortex (superior frontal sulcus) across blocks, and less consistently in parts of the somatosensory and superior parietal cortices. No effects in a control (visual) network Dose dependency: primary motor cortex-dorsal premotor cortex connectivity was significantly higher after iTBS_1800_ compared with iTBS_600_ and iTBS_1200_, but not between iTBS_600_ and iTBS_1200_, and sham Supplementary control experiment: iTBS_1800_ = ↑rsFC between primary motor cortex and bilateral supplementary motor area, dorsal premotor cortex, and parts of the somatosensory and superior parietal cortex when compared to iTBS_600_ followed by 2 sham runs, supporting the notion of a cumulative (dosage) effect, rather than delayed effect of a single dose of iTBS
Nettekoven et al. (2015)	*N* = 16 (7:9) 27.0 ± 3.0	Pretest–posttest, sham-controlled, within-subjects, repeated measures (time-points)	iTBS: 3 runs, 15 min apart 70% RMT	Hot-spot – (L) primary motor cortex; APB	*Resting-state fMRI* **[~ 3 min]** ***Functional connectivity*** In responders, ↑ FC between primary motor cortex and bilateral supplementary motor area and dorsal premotor cortex, as well as the contralateral primary motor cortex rsFC ↑ with each dose of iTBS in responder group, but not for non-responders. i.e. multiple doses of iTBS did not change responsiveness to iTBS (non-responders did not become responders)
Orosz et al. (2012)	*N* = 12 (7:5) 23.9 ± 2.1	Pretest–posttest, within-subjects, sham-controlled	cTBS 80% RMT	Hot-spot – (R) primary motor cortex; small hand muscles	*Task (finger tapping)-based and resting-state fMRI* **[immediate]** ***CBF*** CBF ↑ at (R) primary motor cortex during finger tapping (left hand) compared to rest CBF ↑ at (R) primary motor cortex, and more voxels were implicated compared to baseline
Ruan et al. (2017)	*N* = 60 (30:30) 23.5 ± 4.4	Pretest–posttest, within- and between-subjects	iTBS cTBS cTBS_left_ + iTBS_right_	– (L) primary motor cortex; suprahyoid muscle	*Resting-state fMRI* **[immediate]** ***Functional Connectivity*** cTBS ↓ ALFF in anterior cingulate gyrus iTBS ↑ ALFF to bilateral precuneus cTBS + iTBS ↓ ALFF in brainstem and ↑ ALFF in middle cingulate cortex and (L) precentral gyrus
Ruan et al. (2019)	*N* = 60 (30:30) 23.5 ± 4.4	Pretest–posttest, within- and between- subjects	iTBS cTBS cTBS_left_ + iTBS_right_	– (L) primary motor cortex; suprahyoid muscle	*Resting-state fMRI* **[immediate]** ***Functional Connectivity*** cTBS ↑ ReHo in the (R) lingual gyrus and (R) precuneus, and ↓ ReHo in the posterior cingulate gyrus iTBS ↑ ReHo in the bilateral precentral gyrus, (L) postcentral gyrus, and cuneus, and ↓ ReHo in the (L) cerebellum, brainstem, (L) temporal gyrus, (R) insula, and (L) middle frontal gyrus cTBS + iTBS ↑ ReHo in the precuneus and ↓ ReHo in the (R) cerebellum posterior lobe, (L) cerebellum anterior lobe, and (R) inferior frontal gyrus
Steel et al. (2016)	*N* = 22 (10:12) 26.0 ± 4.2	Within subjects, sham-controlled	cTBS 80% AMT	Hot-spot – (L) primary motor cortex; FDI	*Task (motor procedural learning)- based fMRI* **[330 ± 80 s for the TBS and 450 ± 28 s for sham]** ***BOLD and functional connectivity*** No effects of cTBS on BOLD response at any brain region ↓ global connectivity at (L) primary visual cortex and dorsal premotor area ↑ global connectivity at middle cingulate gyrus, dorsal anterior cingulate, and superior frontal gyrus ↓ FC between (L) inferior occipital gyrus and dorsal premotor area, supplementary motor area, and primary motor cortex ↓ FC between superior occipital gyrus and primary motor cortex and supplementary motor area ↑ FC between the middle temporal and dorsal anterior cingulate ↑ FC between the superior and inferior frontal gyri
Van Nuenun et al. (2012)	*N* = 11 (11:0) 27.0 ± 6.5	Within-subjects, sham-controlled	cTBS 80% AMT	Measurement – (L) dorsal premotor cortex (2 cm anterior and 1 cm medial to hot-spot [FDI])	*Task (cued grip and lift)-based fMRI* **[15 min]** **BOLD** cTBS did not modulate preparatory activity at (L) dorsal premotor cortex
Welniarz et al. (2019)	*N* = 22^c^	Pretest–posttest, within-subjects, sham-controlled	cTBS 90% AMT	Neuronavigation (anatomical) – (R) supplementary motor area	*Task (delayed response cued-movement go-no-go)-based fMRI* **[within 30 min of TBS]** ***BOLD and Functional Connectivity*** cTBS did not affect BOLD Bimanual preparation: FC between (L) supplementary motor area and primary motor cortex was negative before cTBS and positive afterward FC between (L) and (R) supplementary motor area was positive pre cTBS and reinforced post (L) and (R) primary motor cortex FC was positive post cTBS
Zhang et al. (2020)	*N* = 40 (20:20) 23.7 ± 2.6 cTBS: n = 20 (10:10) 23.6 ± 2.2 iTBS: n = 20 (10:10) 23.0 ± 2.7	Pretest–posttest, between-subjects	cTBS iTBS 80% AMT	Hot-spot – (L) primary motor cortex; suprahyoid muscle	*Resting-state fMRI* **[within 30 min]** ***Functional Connectivity*** cTBS: ↑ degree centrality in (L) inferior frontal gyrus compared to baseline iTBS: ↓ degree centrality in the (L) cerebellum and medial frontal gyrus ↑ degree centrality in the (R) superior temporal gyrus, superior frontal gyrus, and postcentral gyrus, as well as the (L) paracentral lobule compared to baseline
*TBS to the prefrontal cortex*					
Alkhasli et al. (2019)	*N* = 16 (8:8) 27.6 ± 7.0	Pretest–posttest, within-subjects	iTBS 90% RMT 120% RMT	Neuronavigation (anatomical/coordinate) – (L) dorsolateral prefrontal cortex: (Tal = − 45, 45, 35)	*Resting-state fMRI* **[7 min]** ***Functional Connectivity*** Sub threshold stimulation: ↑ rsFC between (L) dorsolateral prefrontal cortex and the striatum, (L) and (R) caudate Supra-threshold stimulation: ↑ rsFC between (L) caudate and the (L) and (R) amygdala
Anderkova et al. (2018)	*N* = 20 (7:13) 25.2 ± 2.7	Pretest–posttest, within-subjects	cTBS iTBS 80% AMT	Neuronavigation (anatomical/coordinate) **– (R) inferior frontal gyrus (MNI = 46, 14, 32)** – (L) superior parietal lobule (MNI = − 24, − 68, 48)	*Resting-state fMRI* **[immediate]** ***Functional connectivity*** No reported effects of (R) inferior frontal gyrus stimulation
Gratton, et al. (2013)	*N* = 27 (16:11) 18–31 years ^b^	Pretest–posttest, within-subjects	cTBS 80% AMT	Neuronavigation (anatomical and functional) – (L) dorsolateral prefrontal cortex – (L) anterior insula/frontal operculum – [(L) primary somatosensory cortex]	*Resting-state fMRI* **[~ 10 min]** ***BOLD and Functional Connectivity*** Non-significant trend towards increased rsFC of the fronto-parietal network following TBS to both the (L) dorsolateral prefrontal cortex and (L) anterior insula/frontal operculum compared to active control. Strongest ~ 20 min post TBS No whole brain effects immediately following TBS to either test site. Widespread frontal, parietal and cingulate effects 20 min post stimulation (L) anterior insula/frontal operculum: ↑ FC between (L) anterior insula/frontal operculum and bilateral regions of lateral inferior and middle frontal gyrus and at the (R) intraparietal sulcus, (R) superior frontal gyrus, orbito frontal cortex, posterior temporal lobe, and (L) anterior temporal lobe ↑ FC between (L) dorsolateral prefrontal cortex and anterior and posterior cingulate cortex, bilateral, supramarginal/angular gyrus, bilateral superior frontal gyrus, (L) precentral gyrus, (R) inferior anterior insula, and the midcingulate ↑rsFC between (L) dorsolateral prefrontal cortex and DMN regions (L) dorsolateral prefrontal cortex:↑ rsFC between (L) dorsolateral prefrontal cortex and bilateral areas of the anterior insula/frontal operculum, anterior cingulate, medial superior frontal gyrus, (R) middle frontal gyrus and anterior superior frontal gyrus ↑ rsFC between anterior insula/frontal operculum and (L) dorsal premotor cortex, middle and superior frontal gyrus, angular gyrus, (L) middle and superior temporal gyri and the right supramarginal gyrus No changes after cTBS to primary somatosensory cortex
Gratton et al. (2014)	*N* = 27 (16:11) 18–31 years^b^	Pretest–posttest, within-subjects	cTBS 80% AMT	Neuronavigation (anatomical and functional) – (L) dorsolateral prefrontal cortex – (L) anterior insula/frontal operculum – [(L) primary somatosensory cortex]	*Resting-state fMRI* **[~ 10 min]** **CBF** Trend level ↑ in CBF at site of stimulation following cTBS, variability in direction and magnitude of CBF change following cTBS (increased and decreased) at all sites ↑CBF related to decreased FC of cingulo-opercular or fronto-parietal networks following cTBS to (L) anterior insula/frontal operculum or (L) dorsolateral prefrontal cortex, respectively (not due to underlying baseline relationship) When stratified based on directionality of perfusion, group with ↑ perfusion showed reduced network connectivity, and ↓ perfusion showed increased network connectivity
Gann et al. (2021a)	*N* = 19 (7:12) 22.42 ± 2.36	Pretest-postest, within subjects	iTBS cTBS 80% AMT	Neuronavigation (functional) – (L) dorsolateral prefrontal cortex	*Task (serial reaction time task)- based fMRI* ***[*immediate*]*** ***BOLD and functional connectivity*** No effect of stimulation type on BOLD response in predefined ROI (basal ganglia, hippocampus, dorsolateral prefrontal cortex), no task-related interactions iTBS: ↑BOLD at intraparietal sulcus, cerebellar lobule and frontal cortex during sequence learning (vs random) compared to cTBS ﻿dorsolateral prefrontal cortex-hippocampal FC ↓ as a function of learning (serial reaction time task after stimulation) cTBS: DLPFC-hippocampal FC ↑ as a function of learning (serial reaction time task after stimulation)
Gann et al. (2021b)	*N* = 19 (7:12) 22.42 ± 2.36	Pretest-postest, within subjects	iTBS cTBS 80% AMT	Neuronavigation (functional) – (L) dorsolateral prefrontal cortex	*Resting-state and task (serial reaction time task)- based fMRI* ***[*immediate*]*** ***BOLD – pattern similarity and multivoxel correlation structure*** No effect of c/iTBS affected early- or late-stage pattern similarity change at the dorsolateral prefrontal cortex cTBS: task (sequential, random learning) x stimulation (i/cTBS) interaction indicative of cTBS induced ↓ pattern similarity at early- and late-stage learning/practice during sequential learning at the putamen ↓ resting-state pattern similarity at hippocampus
Hartwigsen et al. (2013)	*N* = 17 (7:10) 23.8 ± 2.2	Within-subjects, sham-controlled	cTBS 80% AMT	Neuronavigation (anatomical/coordinate) – (L) anterior inferior frontal gyrus (MNI = − 52, 34, − 6) – (L) posterior inferior frontal gyrus (MNI = − 52, 13, 8)	*Task (speech production)-based fMRI* **[immediate]** ***BOLD and Functional Connectivity*** (L) posterior inferior frontal gyrus: ↓ BOLD at (L) posterior inferior frontal gyrus and ↑ BOLD at (R) posterior inferior frontal gyrus during pseudo word repetition (L) posterior inferior frontal gyrus: ↑ FC between (R) and (L) posterior inferior frontal gyri
Heinen et al. (2017)	*N* = 16 (10:6) 19–34 years^b^	Pretest–posttest (1^st^ session only), within-subjects, sham-controlled	cTBS 80% AMT	Neuronavigation (anatomical/visual) – (R) frontal eye field (mean MNI = 27, 3, 57)	*Task (visuospatial attention shifting)-based fMRI* **[immediate: 5–10 min]** ***BOLD and Functional Connectivity*** ↓ BOLD bilateral frontal eye field, bilateral supramarginal gyri, (R) inferior parietal lobule, (R) and superior parietal lobule ↓ FC between (R) frontal eye field and (R) supramarginal gyrus, and putamen
Howard et al. (2020)	TBS *n* = 28 (12: 16) 24.0 ± 3.5 SHAM *n* = 28 (12: 16) 24.0 ± 4.5	Between-subjects, sham-controlled	cTBS 80% RMT	Neuronavigation (anatomical/coordinate) – (R) ventrolateral prefrontal cortex (MNI = 48, 38, 20)	*Resting-state fMRI* **[immediate]** ***Functional Connectivity*** ↓ in (R) central/lateral orbitofrontal cortex related global connectivity with cingulate cortex, lateral prefrontal cortex, posterior parietal cortex, ventro-temporal cortex, and left orbitofrontal cortex
Iwabuchi et al. (2017)	*N* = 28^b^ 25.1 ± 7.1	Within-subjects, sham-controlled	iTBS^c^: 3 runs, 5 min apart 80% RMT	Neuronavigation (functional) – (L) dorsolateral prefrontal cortex	*Resting-state fMRI* **[immediate]** ***Functional connectivity*** ↓ between (L) dorsolateral prefrontal cortex and anterior cingulate cortex
Mastropasqua et al. (2014)	TBS: n = 18 (9:9) 26.7 ± 3.8 SHAM: n = 14 (6:8) 27.07 ± 3.6	Pretest–posttest, between-subjects, sham-controlled	cTBS 80% AMT	10–20 system – (R) dorsolateral prefrontal cortex (F4)	*Resting-state fMRI* **[immediate]** ***Functional Connectivity*** ↓ rsFC between (L) dorsolateral prefrontal cortex and (R) posterior parietal cortex
Singh et al. (2020)	*N* = 26 (17:9) 28 ± 8	Pretest–posttest, between-subjects, sham-controlled	iTBS 80% RMT	Neuronavigation (functional) – (L) dorsolateral prefrontal cortex	*Resting-state fMRI* **[10 min]** ***Functional connectivity*** ↑ rsFC of the rostral anterior cingulate cortex 10–15 minuites post stimulation ↓ rsFC between rostral and dorsal anterior cingulate cortices, 27–32 min post iTBS compated to 10–15 minuites post Stronger ↓ in rsFC between rostral and dorsal anterior cingulate cortices, medial prefrontal cortex and frontal poles 45–50 min post stimulation No effects of sham
Shang et al. (2019)	*N* = 36 (15:21) 22.9 ± 3.3	Pretest–posttest, within- and between-subjects, sham-controlled	cTBS 80% RMT	Neuronavigation (anatomical/coordinate) – (L) dorsolateral prefrontal cortex (MNI = − 40, 26, 37)	*Resting-state fMRI* **[immediate]** **Functional Connectivity and CBF** ↓ rsFC between (L) dorsolateral prefrontal cortex and (R) parahippocampal gyrus, (L) lingual gyrus and posterior cingulate cortex/precuneus No effects of sham No local effects on dorsolateral prefrontal cortex activity ↑ CBF to (L) parahippocampal gyrus, (L) hippocampus, (L) amygdala, (L) inferior temporal cortex, (L) inferior parietal cortex and (L) precuneus—this did not survive statistical controls
Tang et al. (2019)	*N* = 10 (6:4) 25.5 ± 2.8	pretest–posttest, within-subjects	iTBS 80% RMT	Neuronavigation (anatomical/coordinate) – (L) dorsolateral prefrontal cortex (MNI = -44, 36, 20)	*Resting-state fMRI* **[immediate, and repeated at 15 min]** **Functional Connectivity** Immediately following TBS: rsFC ↑ between (L) dorsolateral superior frontal gyrus and (L) dorsal inferior frontal gyrus, and ↑ between the (L) rostral inferior frontal gyrus and (R) middle frontal gyrus rsFC ↓ within orbital gyrus regions Effects were attenuated ~ 15 min post TBS 15 min post TBS: rsFC ↓ between caudal inferior frontal gyrus and (R) medial amygdala rsFC ↓ between (L) left caudal inferior frontal gyrus and (R) medial orbital gyrus rsFC ↓ between the (R) opercular inferior frontal gyrus and (L) medial orbital gyrus rsFC ↑ between middle frontal gyrus and (L) orbital gyrus fALFF ↑ at (L) medial superior frontal gyrus, (L) dorsal middle frontal gyrus, (L) ventral cingulate gyrus, and (L) opercular inferior frontal gyrus
Van Holstein et al. (2018)	*N* = 27 (14:13) 21.7 ± 2.0	Within-subjects, no TMS baseline (either pretest, or 30 min post)	cTBS 80% AMT	Neuronavigation (anatomical/coordinate) – (L) anterior prefrontal cortex (MNI = − 30, 60, 8) − (L) dorsolateral prefrontal cortex (MNI = -36,36, 20) − (L) premotor cortex (MNI = − 28, 10, 66)	*Task (task-switching reward manipulation)-based fMRI* **[immediate]** ***BOLD*** (L) anterior prefrontal cortex: non-significant trend towards ↓ reward-related processing in the caudate nucleus No effects at other sites
Vidal-Piñeiro et al. (2014)	*N* = 24 (12:12) 71.8 ± 6.8	Between-subjects, sham-controlled	iTBS 80% AMT	Neuronavigation (anatomical/coordinate) – (L) inferior frontal gyrus (MNI = − 42,14,30)	*Task (encoding memory)-based and resting-state fMRI* **[immediate]** ***BOLD and functional connectivity*** iTBS did not have any effects on rsFC during deep encoding BOLD ↑ at primary visual areas, lateral occipital cortex, ventral occipitotemporal areas and the cerebellum Frontal and posterior (cerebellum-occipital) connectivity was greater during deep encoding post iTBS
Wawrzyniak et al. (2017)	*N* = 20 (10:10) 25.1 ± 2.5	Within- subjects, sham-controlled	cTBS 80% AMT	Neuronavigation (anatomical/coordinate) – (L) anterior inferior frontal gyrus (MNI = − 54, 26, 4) – (L) posterior middle temporal gyrus (MNI = − 51, − 31, 4)	*Resting-state fMRI* **[8.9 ± 0.4 min]** ***Functional Connectivity*** No effects on rsFC
*TBS to the parietal cortex*					
Abellaneda-Pérez et al. (2019)	Younger: *n* = 24 (5:19) 23.4 ± 1.6 Older: *n* = 28 (6:22) 68.2 ± 4.6	Pretest–posttest, between-subjects, sham-controlled	iTBS Younger = 80% AMT Older = 90% AMT	Neuronavigation (functional connectivity) – (L) inferior parietal lobule	*Resting-state fMRI* **[Younger: 33 ± 3 min, Older: 34 ± 5 min]** ***Functional Connectivity*** ↑ rsFC between target and anterior (medial frontal) DMN seeds in younger adults ↑ rsFC between (L) inferior parietal lobe and posterior cingulate cortex in older adults who received active, but not sham iTBS ↑ pre-iTBS rsFC predicted “younger” or “younger like” response to iTBS
Anderkova et al. (2018)	*N* = 20 (7:13) 25.2 ± 2.7	Pretest–posttest, within-subjects	cTBS iTBS 80% AMT	Neuronavigation (anatomical/coordinate) – (R) inferior frontal gyrus (MNI = 46, 14, 32), **– (L) superior parietal lobule (MNI = ** **− 24, − 68, 48)**	*Resting-state fMRI* **[immediate]** ***Functional connectivity*** iTBS to (L) superior parietal lobule: ↑ rsFC between (L) superior parietal lobule and (L) cerebellar nodule, and overall ↑ in rsFC within the dorsal attention network No effects of cTBS
Hermiller et al. (2019)	*N* = 24 (10:14) 23.5 ± 2.6	Within-subjects, sham-controlled	cTBS iTBS 80% RMT	Neuronavigation (functional connectivity) – (L) parietal cortex	*Resting-state fMRI* **[~ 6 min]** ***Functional Connectivity*** No effects of cTBS or iTBS on hippocampal-cortical network (target network), dorsal attention network (control), or primary visual network (control) Relationship between behavioural performance on an episodic memory task and hippocampal-cortical network connectivity
Mancini et al. (2017)	*N* = 15 (7:8) 26 ± 3.28	Pretest–posttest, between-subjects, sham-controlled	cTBS distance adjusted motor threshold	Neuronavigation (anatomical/coordinate) – precuneus (midline)	*Resting-state fMRI* **[5 min]** ***Graph Analysis/Functional Connectivity*** Graph analysis: ↓ involvement of (L) temporal pole at 5–14 min post stimulation. No effects at 15–24 min post stimulation ↑ size of precuneus module at 15–24 min post stimulation Seed-based analysis: ↓ rsFC between precuneus and (L) temporal pole at 5–14 and 15–24 min post stimulation
Thakral et al. (2020)	*N* = 19 (5:14) 21.2 ± 0.38	Pretest–posttest, within-subjects, active-controlled	cTBS	Neuronavigation (functional) – (L) angular gyrus [vertex]	*Resting-state fMRI* **[time to return to scanner unclear/not reported]** ***Functional connectivity*** ↓ functional connectivity between angular gyrus and hippocampal seeds following cTBS to angular gyrus, but not vertex *Note: analyses not fitting specified inclusion criteria have not been reviewed*
Valchev et al. (2015)	*N* = 17 (11:6) 20.9 ± 2.0	Within-subjects, sham-controlled	cTBS 80% RMT	Neuronavigation (functional) – (L) primary somatosensory cortex (mean MNI: − 43 − 35 57)	*Resting-state fMRI* **[within 6 min]** ***Functional connectivity*** ↓ rsFC between (L) primary somatosensory cortex and dorsal premotor cortex, and premotor cortex/supplementary motor area
Valchev et al. (2016)	*N* = 17 (11:6) 20.9 ± 2.0	Within-subjects, sham-controlled	cTBS 80% RMT	Neuronavigation (functional) – (L) primary somatosensory cortex (mean MNI: − 43 − 35 57)	*Task (action/observation)- based fMRI* **[within 6 min]** ***BOLD*** No group effects at target site. Individual results indicate reduction of signal for some participants, and an increase for others
*TBS to the temporal cortex*					
Andoh et al. (2013)	N = 13 (6:7) 23.3 ± 5.9	Pretest–posttest, within-subjects	cTBS 41% MSO	Neuronavigation (functional activation): – (L) anterolateral Heschl's gyrus – (R) anterolateral Heschl's gyrus Neuronavigation (anatomical) – [vertex]	*Task (melody)-based fMRI* **[immediate: 2.8 min ± 0.4 min]** ***BOLD and Functional Connectivity*** (R) anterolateral Heschl's gyrus: ↑ BOLD at (R) anterolateral Heschl's gyrus, inferior and superior temporal cortices, and middle frontal gyrus (R) anterolateral Heschl's gyrus: ↑ FC between (L)/(R) auditory cortices, (L) anterolateral Heschl's gyrus and (R) pre- & post- central gyri and insula
Andoh et al. (2015)	*N* = 17 (8:9) 23.1 ± 4.9	Pretest–posttest, within-subjects	cTBS 41% MSO	Neuronavigation (Anatomical/coordinate) – (L) anterolateral Heschl's gyrus (MNI = -51.4, -17.2, 2.6) – (R) anterolateral Heschl's gyrus (MNI = 54.6, -10.8, 0.3) – [vertex (anatomically defined)]	*Resting-state fMRI* **[immediate: day 1 = 2.8** ± **0.5 min, day 2 = 2.5** ± **0.3 min, day 3 = 2.4** ± **0.1 min]** ***Functional Connectivity*** (R) anterolateral Heschl's gyrus: ↓ in ipsilateral and contralateral auditory regions, and bilateral motor (including motor, premotor, and primary and secondary somatosensory cortices) regions (L) anterolateral Heschl's gyrus: ↓ rsFC with (R) anterolateral Heschl's gyrus
Pitcher et al. (2014)	*N* = 15^b^	Pretest–posttest, within-subjects	cTBS; Total pulses: 900 80% AMT or 30% MSO (whichever was higher)	Neuronavigation (functional) **– (R) posterior superior temporal sulcus** – (R) occipital face area	Task* (face/emotion processing)-based fMRI* **[time to return to scanner unclear/not reported]** ***BOLD*** (R) posterior superior temporal sulcus: ↓ (R) posterior superior temporal sulcus (dynamic faces)
Pitcher et al. (2017)	*N* = 23 (10:13)^b^	Pretest–posttest, within-subjects	cTBS; Total pulses: 900 80% AMT or 30% MSO (whichever was higher)	Neuronavigation (functional) – (R) posterior superior temporal sulcus – [vertex]; top of the head halfway between nasion/inion	*Task (face/emotion processing)-based fMRI* **[immediate]** ***BOLD*** ↓ BOLD in response to faces at (R) posterior superior temporal sulcus, (R) anterior posterior superior temporal sulcus, and amygdala
Soutschekid et al. (2020)	*N* = 60 (23:37) 23.4 ± 2.4	Pretest–posttest, between-subjects, active-controlled	cTBS 80% AMT	Neuronavigation (anatomical/coordinate) – (R) temporoparietal junction (MNI = 60, − 58, 31) [vertex]	*Task (delayed gratification)-based fMRI* **[immediate]** ***BOLD and Psychophysiological interactions (connectivity)*** No effects on striatum or ventromedial prefrontal cortex based on region of interest analysis. Interaction between delayed gratification and connectivity between the (R) temporoparietal junction and striatum ↓ dorsolateral prefrontal cortex activation following cTBS to the (R) temporoparietal junction compared to vertex, but no difference in (R) temporoparietal junction—dorsolateral prefrontal cortex connectivity between (R) temporoparietal junction and vertex stimulation Exploratory whole brain analysis revealed no effect of cTBS during task performance
*TBS to the occipital cortex*					
Groen et al. (2021)	*N* = 16 (4:12) Average age = 24.4 years	Pretest–posttest, within-subjects, sham- and active-controlled	cTBS 30% MSO	Neuronavigation (functional) – (R) occipital place area – [(R) occipital face area	*Task (scene selectivity)-related fMRI* **[3 min]** **BOLD** ↓ BOLD at parahippacampal face area post active stimulation (both conditions, stronger effects from occipital place area)—no effects of scene type/condition (ROI and whole brain analysis) ↓ BOLD at fusiform face area following active control (occipital face area) stimulation—no effects of scene type/condition (ROI and whole brain analysis) ↓ BOLD at occipital place area, fusiform face area, occipital face area and parahippacampal face area post occipital place area stimulation no effects of scene type/condition (whole brain analysis) Occipital face area stimulation resulted in ↑ BOLD at occipital face area, and ↓ BOLD at occipital place area, fusiform face area and parahippocampal face area—no effects of scene type/condition (whole brain analysis)
Pitcher et al. (2014)	*N* = 15^b^	Pretest–posttest, within-subjects	cTBS; Total pulses: 900 80% AMT or 30% MSO (whichever was higher)	Neuronavigation (functional) – (R) posterior superior temporal sulcus **– (R) occipital face area**	Task* (face/emotion processing)-based fMRI* **[time to return to scanner unclear/not reported]** ***BOLD*** (R) occipital face area: ↓ (R) posterior superior temporal sulcus (static faces)
Rahnev et al. (2013)	*N* = 4(2:2) 23–32 years^b^	Pretest–posttest, within-subjects	cTBS 80% phosphene threshold	Hot-spot “hunting procedure” – (L) occipital cortex – [vertex]	*Resting-state fMRI* **[time to return to scanner unclear/not reported]** ***Functional connectivity*** ↓ between V1-2, V1-3, V2-3 ↓ between L-R V1, V2, V3
*TBS to the cerebellum*					
Halko et al. (2014)	*N* = 9 (5:4)^b^	Pretest–posttest, within-subjects, sham-controlled	iTBS 100% AMT	Neuronavigation (functional) – (R) lateral cerebellum; Crus I or Crus II (mean MNI = 41, 72, 39) Neuronavigation (anatomical) – midline cerebellum; lobule VII (MNI = 1, 73, 33)	*Resting-state fMRI* **[immediate]** ***Functional Connectivity*** (R) lateral cerebellum: ↑ DMN FC Medial cerebellum: ↑ dorsal attention network connectivity
Odorfer et al. (2019)	*N* = 8^b^	Pretest–posttest, between-subjects	cTBS 80% AMT	Measurement (3 cm lateral and 1 cm inferior to the inion) – (L) cerebellum (lobule VIII) followed by (R) cerebellum (60 s break between sites) – [dorsal premotor cortex]	*Task (finger-tapping)- related fMRI* **BOLD** **[immediate]** Cerebellar cTBS had no effects on brain activation in healthy controls
Rastogi et al. (2017)	N = 12 (7:5) 29.7 ± 9.4	Pretest–posttest, within-subjects, sham-controlled	cTBS 80% AMT	Measurement – (R) cerebellum; crus 1 (1 cm inferior and 3 cm to the right of the inion)	*Resting-state fMRI* **[immediate]** ***Functional Connectivity*** ↓ rsFC in active compared to sham cTBS in non-motor (cognitive) network: (L) inferior parietal lobe, posterior medial frontal cortex, lateral prefrontal cortex, and (R) medial posterior parietal cortex (precuneus) No effect on motor network

↑: increased, ↓: decreased, *L*: left, *R*: right, *ALFF* amplitude of low-frequency fluctuation, *AMT* active motor threshold, *APB* abductor pollicis brevis, *BOLD* blood oxygen level dependant, *CBF* cerebral blood flow, *cTBS* continuous theta burst stimulation, *DMN* default mode network, *fALFF* functional amplitude of low-frequency fluctuation, *FC* functional connectivity, *FDI* first dorsal interosseous, *fMRI* functional magnetic resonance imaging, *iTBS* intermittent theta burst stimulation, *MNI* Montreal Neurological Institute coordinate system, *MSO* maximum stimulator output, *ReHo* regional homogeneity, *RMT* resting motor threshold, *ROI *region of interest, *rsFC* resting-state functional connectivity, *TBS* theta burst stimulation

^a^Parameter detail only provided when divergent from those reported by Huang et al. (2005). Where detail was not provided, it has been assumed that the protocol is comparable to that reported by Huang et al. (2005)

^b^Demographic information unclear, incomplete or not provided

^c^Authors (Iwabuchi et al. 2017) report iTBS, however, parameters are consistent with cTBS

## Discussion

This review sought to systematically synthesise and evaluate the fMRI literature investigating the functional neurobiological aftereffects of TBS applied to the human brain in neurotypical adults. TBS-induced alterations in offline brain activity and connectivity are summarised herein. In all reviewed studies, TBS was applied at rest, i.e., offline and in the absence of any cognitively demanding tasks or stimuli. As shown in Table 2, the response to both TBS protocols, as measured by fMRI, is variable. Therefore, outcomes will not be summarised in line with the generally “expected” responses to TBS described in the introduction. Instead, the relevant literature will be consolidated based on target regions. Outcomes measured at rest (i.e., in the absence of any cognitively or behaviourally demanding stimuli) and during offline task completion will be considered separately, as these contextual factors have neurobiological implications. Themes and patterns emerging from this summary which help to elucidate the observed variability will then be discussed.

### Functional neurobiological responses to TBS across the cortex

#### TBS to the motor cortex

We first summarise studies reporting fMRI outcomes of TBS applied to motor sites, as most available TMS knowledge comes from research targeting this region. In total, 17 of the identified studies targeted motor regions.

At rest, neither cTBS to the left supplementary motor area (SMA) (Ji et al. 2017) nor iTBS to the left M1 (Cárdenas-Morales et al. 2011) induced any measurable effects on blood oxygen level dependant (BOLD) response or cerebral blood flow (CBF), respectively. Matsuta et al. (2022) further add that cTBS to the left motor representation of the abductor pollicis brevis (APB) had no consequences on resting state network activity, including the default mode network (DMN) and primary motor network. Regarding resting-state functional connectivity ([rs]FC) however, cTBS to the M1 hand (Hu et al. 2017) and suprahyoid muscle (Ruan et al. 2017, 2019) representations, as well as the SMA (Ji et al. 2017, 2020) resulted in reduced network rsFC. Timing of post-stimulation follow-ups might, however, be a critical factor. For example, immediately post cTBS, Ji et al. (2020) report reduced rsFC of the bilateral cerebellum. Conversely, a second post-cTBS fMRI run indicated increased rsFC of the bilateral paracentral gyri. In contrast, iTBS to the M1 hand (Nettekoven et al. 2014, 2015) or suprahyoid muscle (Ruan et al. 2017, 2019) representations generally increased rsFC, though some conflicting findings are also reported (Cárdenas-Morales et al. 2011; Zhang et al. 2020). Zhang et al. (2020) report increased degree centrality (DC), a graph-based approach for investigating rsFC, in the left inferior frontal gyrus (IFG) following cTBS to the left suprahyoid M1 representation. In contrast, iTBS to this site resulted in both increased (at the superior temporal gyrus, right superior frontal gyrus, right postcentral gyrus, and left paracentral lobule) and decreased (at the left cerebellum and left medial frontal gyrus) DC.

Despite no effects of iTBS to M1 on rsFC outcomes (Cárdenas-Morales et al. 2011), during a choice reaction task, Cárdenas-Morales and colleagues report decreased BOLD responses at numerous motor and parietal sites (Cárdenas-Morales et al. 2011). cTBS to various motor regions has been shown not to affect BOLD response during components of task/behaviour related aspects of motor performance that might be considered to have cognitive underpinnings, such as motor procedural learning (M1 hand representation; Steel et al. 2016), preparatory phases of a motor action (dorsal premotor cortex; van Nuenen et al. 2012), and response delays (SMA; Welniarz et al. 2019). Conversely, yet still relevant to cognitive processes, following cTBS to the ventral premotor cortex (PMv) Agnew and colleagues (2018) reported increases and decreases in BOLD response at frontal, motor, parietal, and subcortical regions during emotion processing, but no local effects of stimulation. Finally, when accompanied by nociceptive (via gaseous CO_2_) stimulation, cTBS to M1 reduced BOLD activity at the rolandic operculum, insula and postcentral gyrus. The strength of the relationship between BOLD signal and stimulus strength was also reduced (Annak et al. 2019).

During motor execution, left-handed finger tapping following cTBS resulted in increased CBF at the targeted right M1. The authors also reported activation of a larger area of M1 compared to baseline (Orosz et al. 2012). In line with these findings, Cocchi et al. (2015) also observed facilitatory effects of cTBS applied to the right M1. Even at rest, the authors found increased responsiveness of non-motor regions involved in the production of left thumb movements, which was a function of specialisation for the targeted right APB motor cortex representation (i.e., “hot-spot”), including the insula, striatum, and left temporal cortex.

Irrespective of the exact stimulation location, network-wide increases in rsFC were observed mainly in response to motor iTBS, and network-wide reductions in rsFC were reported in response to cTBS, though some inconsistency was noted. The introduction of cognitively or physically demanding tasks yielded more inconsistent results, and the specific task or target behaviour, also appears to mediate responses. We speculate that this might, at least in part, reflect the complexity of networks involved in associated processes. Another important consideration, however, is that while most studies investigating resting-state outcomes targeted M1 regions, the exact location/motor representation varied. Regarding task-related effects, less literature was available, and there was even more variability in the motor regions targeted.

#### TBS to the prefrontal cortex

Outside of the motor cortex, various regions of the prefrontal cortex (PFC) were commonly investigated using TMS protocols. In this review we identified 17 studies targeting various prefrontal brain regions. Given the diversity of prefrontal targets, where possible, we attempted to synthesise these as focally as practicable.

The dorsolateral prefrontal cortex (DLPFC) is perhaps the most commonly targeted frontal region for TMS research. This is due to its critical involvement in numerous cognitive processes (Balconi 2013; Brunoni and Vanderhasselt 2014), its well-established role as an efficacious clinical target for major depressive disorder (Perera et al. 2016), having been trialled as a potential target for numerous conditions with neurobiological underpinnings (Doruk Camsari et al. 2018), and ease of access. The effects of TBS to the DLPFC as measured by fMRI, however, are inconsistent. At rest, network-wide increases (Gratton et al. 2013, 2014; Shang et al. 2019) and decreases (Iwabuchi et al. 2017; Mastropasqua et al. 2014; Shang et al. 2019) in rsFC and CBF have been noted in response to cTBS. While less research has investigated the neurobiological effects of iTBS to the DLPFC, again, at rest, both network-wide increases (Alkhasli et al. 2019; Tang et al. 2019) and decreases (Tang et al. 2019) have been observed. Singh et al. (2020) provide some evidence that these effects might be time-dependant. Specifically, the authors report increased DMN FC 10–15 min post iTBS to the left DLPFC, while at two consecutive time-points, 27–32 and 45–50 min respectively, FC of the DMN decreased and became more widespread. While not targeting the DLPFC specifically, Howard and colleagues (Howard et al. 2020) applied TBS to a ventral region of the right lateral PFC, to indirectly modulate activity at the orbitofrontal cortex (OFC). At rest, cTBS to this region resulted in widespread reductions in global connectivity of the right central/lateral OFC network (Howard et al. 2020).

One study investigated the effects of cTBS to the left DLPFC on neurobiological responses to an offline task (switching protocol with a reward manipulation to investigate reward anticipation [motivation], and task [cognitive] or response [action] switching performance) (Van Holstein et al. 2018). The authors reported no significant effect of stimulation on BOLD responses at the target region, or distal brain regions (Van Holstein et al. 2018). Gann et al. (2021a) similarly identified no effects of either iTBS or cTBS to the left DLPFC on their predefined ROIs, including the: basal ganglia, hippocampus, and DLPFC, during a learning (serial reaction time task) paradigm. Following iTBS, compared to cTBS however, more widespread effects were noted by way of increased BOLD at the intraparietal sulcus, cerebellar lobule and frontal cortex during sequence (compared to random) learning. Furthermore, motor sequence learning increased fronto-hippocampal FC following cTBS, while reduced fronto-hippocampal FC was observed following iTBS. The authors (Gann et al. 2021a) attribute this finding to a cTBS induced disruption to typical processing, whereby learning itself reduces fronto-hippocampal connectivity. In a related study by the same group, (Gann et al. 2021b) prefrontal stimulation had no significant impact upon early- and late-stage learning-related DLPFC response patterns, for which greater differences were observed during sequence versus random learning.

Prefrontal cTBS did, however, affect similarity patterns of early/late stage learning-related activity of the putamen, whereby less similarity was observed between early- and late-stage learning/practice for sequential, rather than random, learning (Gann et al. 2021b). Prefrontal TBS also affected pre- and post-stimulation hippocampal resting-state pattern similarity, again, similarity was reduced as a function of cTBS (Gann et al. 2021b).

The IFG has also been a target of interest for the TBS research identified in this review. While some authors report no effects of iTBS (Anderkova et al. 2018) nor cTBS (Wawrzyniak et al. 2017; Anderkova et al. 2018) to the IFG on rsFC, during task performance, the effects of both TBS protocols have been observed. Specifically, iTBS to the left IFG resulted in widespread increased network-wide activity and connectivity at frontal, occipital and cerebellar regions during phases of encoding in older adults (Vidal-Piñeiro et al. 2014). During pseudoword repetition, task-related BOLD response was reduced at the target left IFG following cTBS, and increased at the contralateral homologue (Hartwigsen et al. 2013).cTBS has also been shown to increase rsFC and CBF when applied to the anterior insula/frontal operculum (Gratton et al. 2013, 2014), and decrease network-wide BOLD response and connectivity during an attention shifting paradigm when applied to the frontal eye field region (Heinen et al. 2017). As only single studies have targeted these regions, no interpretations can be made.

Unlike the pattern of results described regarding stimulation to the motor cortex, frontal stimulation yields more inconsistent findings. There is evidence of both facilitatory and inhibitory responses to both TBS protocols at rest and during various tasks. This apparent inconsistency in response to TBS effects, therefore, appears to go beyond task- or state-dependence when the PFC is targeted. Several factors may contribute to this variability. For example, different approaches to target, specifically DLPFC, localisation (Rusjan et al. 2010) and coil position/angle (Tsuyama et al. 2009), can affect stimulation outcomes in this region. Other possible considerations regarding these findings are the role that the complexity of neural organisation of the frontal cortex has in producing these effects (Kolb et al. 2012), or the complexity of cognitive and behavioural demands associated with the tasks performed.

#### TBS to the parietal cortex

Seven studies applied TBS to the parietal cortex. iTBS to the left inferior parietal lobe (IPL) (Abellaneda-Pérez et al. 2019) and superior parietal lobe (Anderkova et al. 2018) has been demonstrated to increase network-wide rsFC (Abellaneda-Pérez et al. 2019; Anderkova et al. 2018).While stimulating the IPL, Anderkova et al. (2018) report that cTBS did not produce any significant effects on neurobiological function at rest. Conversely, three studies reported reduced rsFC following cTBS to the angular gyrus (Thakral et al. 2020), left somatosensory cortex (Valchev et al. 2015), and precuneus (Mancini et al. 2017). Interestingly, cTBS to the precuneus also increased the spread of activity at this region 15–24 min post stimulation. When the left parietal cortex was targeted based on connectivity with the hippocampal-cortical network (HCN), Hermiller et al. (2019) reported no effects of either TBS protocol on the target HCN, or on the control dorsal attention network (DAN) or primary visual network. The effects of cTBS to parietal regions of the brain on the neural basis of offline task performance has only been investigated by one study reviewed here, which reported no effects of cTBS to the left somatosensory cortex on action/observation task performance (Valchev et al. 2016). Based on the limited research targeting the parietal cortex, it appears that the neurobiological effects of parietal iTBS are more readily observable than those of cTBS. There are, however, too few studies to enable solid rationales for this outcome to be determined.

#### TBS to the temporal cortex

A total of five identified studies applied TBS to areas of the temporal cortex. Two studies, from the same group, targeted the auditory cortex, specifically, anterolateral Heschl’s gyrus (HGal). cTBS to right HGal reduced rsFC in ipsilateral and contralateral auditory regions, and in bilateral motor and somatosensory (S1 and S2) regions, and cTBS to the left HGal resulted in reduced rsFC between the target region and contralateral homologue (Andoh et al. 2015). A different neurobiological response to cTBS, however, was observed in a task-state (Andoh and Zatorre 2013). During a melody discrimination task, cTBS to the right HGal increased BOLD responses at the contralateral homologue, as well as inferior and superior temporal cortices, and the middle frontal gyrus. rsFC between the left HGal, right pre- and post-central gyri, and the insula also increased. Stimulation to the left HGal elicited no such effects (Andoh and Zatorre 2013). Another two studies, again from the same group, demonstrate that cTBS to the right posterior superior temporal sulcus (pSTS) induced reductions in BOLD response at the target and proximal temporal regions, as well as the amygdala during face emotion processing (Pitcher et al. 2014, 2017). cTBS to a proximal target site, the right temporoparietal junction (TPJ), resulted in reduced DLPFC activation during a delayed gratification experiment. There was no evidence, however, of connectivity between these sites (Soutschekid et al. 2020). The authors did, however, note that delayed gratification, mediated connectivity between the right TPJ and the striatum, despite no effects on BOLD response. Care must be taken when interpreting these findings, as only a small number of studies, and from the same groups, are reviewed here. Again, however, increased variability was observed when task-related outcomes were assessed.

#### TBS to the occipital cortex

cTBS to the left occipital cortex has been shown to reduce rsFC between the primary, secondary, and third visual cortices (V1–V2, V1–V3, and V2–V3) bilaterally (Rahnev et al. 2013), and has also been shown to reduce BOLD response during the presentation of static faces at the right pSTS (Pitcher et al. 2014). cTBS to right lateralised occipital scene selective (place and face) areas has also resulted largely in reduced BOLD activity across various scene and face selective regions, not seemingly affected by stimulus (scene) condition (Groen et al. 2021). Given that only three studies stimulated the occipital lobe using cTBS (and none with iTBS), all measuring different outcomes, no meaningful explanation of the effects of TBS to this region can be provided.

#### TBS to the cerebellum

Cerebellar stimulation was applied in three of the identified studies. cTBS to the right lateral cerebellum (Crus I) decreases rsFC between core DMN regions (Rastogi et al. 2017), whereas iTBS targeting an overlapping region (Crus I/II) increased rsFC within DMN regions (Halko et al. 2014). iTBS to the medial cerebellum, increased DAN rsFC (Halko et al. 2014). During a finger-tapping paradigm, however, cerebellar cTBS was not shown to have effects on brain regions implicated in motor performance during such tasks in healthy populations (Odorfer 2019).

A pertinent consideration regarding cerebellar stimulation, given the location of the cerebellum within the skull and its cellular organisation, is whether the magnetic field created by the TMS coil can adequately and focally stimulate the target site (van Dun et al. 2017). Double-coned coils might be more effective for cerebellar stimulation (van Dun et al. 2017; Fernandez et al. 2018, 2020), though none of the studies reviewed here used this apparatus. The studies which reported using standard figure-of-eight coils, all demonstrated effects of cerebellar TBS (Halko et al. 2014; Rastogi et al. 2017). In contrast, Odorfer (2019), who used a figure-of-eight coil with a slight bend for curved scalp locations (MagVenture, Inc., Georgia, USA) which may improve the depth of penetration to a target site, reported no effects of stimulation.

### Limitations of the reviewed literature and directions for future research

#### Regarding the observed outcomes

While tasks and state were identified as critical factors influencing the fMRI response to TBS, future research needs to characterise these effects explicitly. A greater understanding of the influence of different tasks on neurobiological responses is necessary to aid the selection of the most appropriate TBS protocol. Regarding resting-state protocols, increased consistency in terms of participant actions during, and even immediately before this period, would be beneficial. For example, standardised protocols regarding having eyes open/closed, focusing on a fixation cross versus a dark screen, or being instructed to “mind-wonder” might be implemented. In a similar vein, such parameters *during* stimulation also need to be more precisely controlled and reported. Where possible, both resting-state and task-related outcomes should be collected within the same study.

#### Participant factors

In the broader non-invasive brain stimulation (NIBS) literature, several individual/participant factors have been identified as contributing to the variability in response to stimulation. These include age, biological sex, and genetic/epigenetics (Pellegrini et al. 2018; Ridding and Ziemann 2010), though there are likely many unknown sources of variability also. These influences, however, were seldom investigated or appropriately controlled for in the reviewed literature. Indeed, only one study reviewed here systematically investigated the effects of age (Abellaneda-Pérez et al. 2019), and while most studies reported here include young-middle aged adults, two studies (Abellaneda-Pérez et al. 2019; Vidal-Piñeiro et al. 2014) report samples > 65 years. These studies must be considered with caution as neurodevelopmental factors associated with this population might confound results. Further, while Hu et al. (2017) reported outcomes stratified by biological sex, statistical comparisons were not presented. None of the reviewed literature investigated genetic or epigenetic effects.

More broadly, despite studies often reporting group effects, and the response to TBS being described as having effects in a particular direction, there is evidence from the literature investigating the effects of TBS on MEPs that demonstrates a great deal of individual variability to TBS response. While single pulses of TMS appear to have reasonably consistent effects on MEPs, the effects of TBS are far more variable and difficult to reproduce (Ozdemir et al. 2021).That is, there are reports of some participants showing facilitatory outcomes, others inhibitory, and others classified as “non-responders” within the same study/protocol (Do et al. 2018; Hamada et al. 2013; Goldsworthy et al. 2014; Vallence et al. 2015; Corp et al. 2020; Jannati et al. 2017), and these outcomes can change across sessions (Ozdemir et al. 2021). Of the reviewed studies, only three evaluated or considered inter-individual variability in their own outcomes (Abellaneda-Pérez et al. 2019; Nettekoven et al. 2015; Rahnev et al. 2013). Only Rahnev et al. (2013) report outcomes for individual participants. Despite observing a similar pattern of response across all four participants (i.e. reduced FC between visual regions) these changes did not reach statistical significance for all participants—those for whom the change was not statistically significant might be considered non-responders, though there is no clear and precise criteria for this. Nettekoven et al. (2015) sought to investigate whether applying multiple runs of TBS to increase the dose would transition non-responders, those with < 10% change in MEP size compared to baseline, into responders. While each dose of iTBS further increased connectivity and MEP outcomes among the group defined as responders, resting state and MEP outcomes remained comparable to baseline among the non-responder group, irrespective of dose (Nettekoven et al. 2015). Abellaneda-Pérez et al. (2019), however, report that inter-individual variability might be reduced in older adults following active, compared to sham, stimulation.

There are several factors that contribute to this variability in response to TBS. Age, biological sex, and genetic/epigenetic factors are among the most well documented participant factors that contribute to inter-individual variability in response to TBS, and NIBS more broadly (Pellegrini et al. 2018; Ridding and Ziemann 2010). In addition to these participant factors, the studies reviewed here also point towards underlying anatomical structure (Abellaneda-Pérez et al. 2019; Agnew et al. 2018) and behavioural (Hermiller et al. 2019; Annak et al. 2019) contributors to individual responses to TBS. Beyond this, there is also evidence that the corticospinal state at the onset of stimulation can yield variability in outcomes. Specifically, Zrenner et al. (2018) report that triggering rTMS based on a high-excitability state determined by a negative peak in μ-rhythm resulted in increased corticospinal activity following stimulation, while rTMS triggered at a low-excitability phase, or at random yielded no overall effects. These factors all require further investigation to determine the extent of their impact on TBS outcomes.

#### Methodological factors

Perhaps one of the most common methodological flaws in the neuroscience literature broadly is that of inadequate sample size. Indeed, only nine volunteers participated in the seminal work conducted by Huang et al. (2005), and the largest sample of the reviewed studies comprised 60 participants (Soutschekid et al. 2020). In their survey of researchers engaging in TMS work, Héroux et al. (2015) report that less than a quarter of respondents indicated using formal power calculations to determine their sample size, while others relied on previous experience, or adjusted the sample size depending on the observed effects. None of the reviewed studies reported how sample size was determined. This variability in determining an appropriate sample size might be one critical factor in another fundamental problem with the TMS literature; that of reproducibility of findings. Only 45% of respondents in the survey by Héroux et al. (2015) reported reproducing findings of original TBS research, with many indicating more variability in their outcomes. In a field where high levels of inter-individual variability are now well established, as described previously in this review, the robustness of reported outcomes is called into question. Beyond the statistical appropriateness of power calculations and its potential impact on reproducibility, another (and perhaps more dire) finding reported by Héroux et al. (2015) was that many respondents admitted to knowing of others who engage in, or themselves confessed to having engaged in, questionable research practices. This included screening for participants known to be responders, selective reporting, or rejection of data without justification. Oftentimes these practices are not reported in publications (Héroux et al. 2015). The literature reviewed by the present study is then further restricted by a focus on MRI outcomes only. Small sample size is often considered unavoidable in neuroimaging research due to high costs associated with conducting such research, as well as access to participants and time constraints (Button et al. 2013). Consequently, insufficient power coupled with small effects, and questionable practices reduces the likelihood of detecting true effects and impacts upon reproducibility of both NIBS (Héroux et al. 2015) and neuroimaging (Button et al. 2013) literature. Growing acceptance of this problem in the field of neuroimaging has led towards numerous data sharing initiatives, now widely accessed across many areas. Similar initiatives have already been implemented regarding electrophysiological outcomes of TBS (Corp et al. 2020) and similar practices regarding neuroimaging outcomes in response to TBS, and other NIBS protocols, would be of immense benefit to the field. For such an initiative to be fruitful, however, greater consistency across protocols is imperative.

In terms of protocols, there were several sources of methodological variability across studies which can also influence TBS outcomes. Perhaps one of the most common sources of variability in the TBS literature broadly is the stimulation intensity. In their seminal TBS paper, Huang and colleagues (Huang et al. 2005) applied TBS at 80% of active motor threshold. For the reviewed studies, stimulation intensity ranged between 80 and 100% of active motor threshold, 70–120% of resting motor threshold, or 30–40% of maximum stimulator output. The effects of sub- versus supra-threshold stimulation were investigated by one study (Alkhasli et al. 2019), which demonstrated altered outcomes at different intensities. These results are not readily generalisable to the broader TBS literature, as stimulation is typically administered at sub-threshold intensities, however, point towards intensity-related variability in outcomes.

TBS dose was modulated in two ways: either by manipulating the protocol parameters and, therefore, the number of pulses delivered (Agnew et al. 2018; Pitcher et al. 2014, 2017), or by applying multiple runs of TBS to the same site at timed intervals (Ji et al. 2017, 2020; Nettekoven et al. 2014, 2015), which may induce metaplastic-like effects (Karabanov et al. 2015). One study investigated the impact of TBS dose (Nettekoven et al. 2014), and while a single run of iTBS (iTBS_600_) increased rsFC within a predefined motor network, effects were stronger following three runs of iTBS (iTBS_1800_) compared to just one or two (iTBS_1200_). The observed effects were indeed cumulative effects of iTBS, rather than a delayed response to a single run of iTBS (Nettekoven et al. 2014).

Finally, most studies reported returning participants to the scanner “immediately” following TBS, though many did not specify the time taken to commence the scan. For those who provided this information, the time to return to the scanner ranged between ~ 2 and 30 min across studies. More systematic reporting in this regard is paramount. This inconsistency is likely to affect observed outcomes, as some reported a strengthening of the TBS effects over time (Tang et al. 2019; Gratton et al. 2013), while other report shifts in the direction (Heinen et al. 2017; Hu et al. 2017; Ji et al. 2020) or location (Ji et al. 2020) of response, or a combination of these outcomes (Singh et al. 2020). Further, the duration of time that the TBS effects remain appears site/network dependant. Ji et al. (2017), report that dynamic functional connectivity effects at the targeted left SMA might outlast similar responses observed at other regions (specifically, the IFG).

Such methodological inconsistencies make it difficult to elucidate related effects clearly or to provide reliable directions regarding optimal practices. The available literature does, however, demonstrate that these factors contribute to TBS outcome variability. Other methodological inconsistencies noted in the reviewed literature included: site variability, methods for locating the target site, outcome measures, analysis approaches, and equipment. These, however, were not systematically investigated in any of the reviewed studies, and our review does not provide any clear, consistent, or differentiating effects on outcomes based on these factors. Therefore, no speculations will be made in this regard.

#### Non-specific effects of TBS

Next, we consider the methodological approaches taken when attempting to account for non-specific effects of TBS. Several studies targeted the vertex as an active (Pitcher et al. 2017; Agnew et al. 2018; Andoh et al. 2015; Andoh and Zatorre 2013; Rahnev et al. 2013) or sham (Nettekoven et al. 2014, 2015) control site, while others performed statistical comparisons between the target and a non-target network (Hermiller et al. 2019; Wawrzyniak et al. 2017; Nettekoven et al. 2014; Halko et al. 2014; Rastogi et al. 2017).

The vertex is commonly considered an appropriate control site in TMS research, being referred to by some as an “empty quarter” unlikely to play a role in the target (often behavioural) mechanisms (Davis et al. 2013; Jung et al. 2016). Agnew et al. (2018), however, reported overlapping changes in BOLD response following cTBS to the target right PMv and active-control cTBS applied to the vertex, thus demonstrating non-specific effects of stimulation. This finding is not reflective of the spread or focality of cTBS, but rather of possible network overlap concerning the stimulated regions, highlighting that the vertex should not be considered a blanket control site. An additional caveat of active-control protocols is, depending on the location of the target site, that control stimulation to the vertex can feel and sound different to the target region (Davis et al. 2013), which might reduce its effectiveness as a comparable control. Furthermore, such stimulation might only be appropriate when testing certain outcome measures. For example, 1 Hz rTMS to the vertex reduced BOLD at DMN regions (Jung et al. 2016) and therefore would have implications for resting-state outcomes. As many DMN regions are also involved in cognitive processes, however, careful consideration must be taken when selecting the vertex as an active control site, irrespective of outcome state. Active-control stimulation should target a region unrelated to, or less implicated in, the tasks and mechanisms of interest, e.g. targeting the opposite hemisphere (Andoh et al. 2015; Andoh and Zatorre 2013), or a region implicated in a control, or unrelated, neural network. Indeed, Groen et al. (2021) report largely overlapping effects of cTBS to the occipital place- and face- areas when investigating scene specific neurobiological responses.

Studies comparing effects at target and non-target networks indicate that the effects of TBS are not global (i.e., brain-wide), but instead specifically act on the target network. This has been demonstrated by studies showing TBS-induced modulation of cortical networks involved in cognition, but not cortical motor networks, following stimulation to “cognitive” cerebellar regions (Halko et al. 2014; Rastogi et al. 2017). Similarly, functional connectivity within the visual network, acting as a control network, was not shown to be influenced by iTBS over M1 or the vertex (Nettekoven et al. 2014). Others, however, report no significant neurobiological effects of TBS to either the target or control networks (Hermiller et al. 2019; Wawrzyniak et al. 2017). It is crucial to note that many studies reviewed here report widespread effects of TBS which might go beyond the intended/target region or network of interest. A recent review of resting-state fMRI outcomes of several rTMS protocols, including TBS, also indicates that the effects are not network-specific (Beynel et al. 2020).

No studies directly investigated the effects of active-control stimulation versus comparisons to a non-target network on fMRI outcomes. Each approach might only be appropriate in certain situations, the selection of which requires careful consideration. Task- and state-based factors related to outcome measures are likely to affect the networks and regions modulated by TBS, and therefore must be considered when selecting the most appropriate comparison.

### Consolidation of findings and direction for future research

The response to TBS, as measured using fMRI, appears to be predominantly state- and task-dependent. At rest, the observed response to TBS was generally aligned with the electrophysiological outcomes described by Huang et al. (2005). That is, cTBS appeared to reduce, while iTBS increased rsFC. An important exception to this observed pattern occurred when stimulation was applied to the PFC. Here, considerable variability existed across all outcome measures, irrespective of state or task.

The introduction of external stimuli, by means of cognitive or behavioural tasks, greatly affects the neurobiological response to TBS, and vastly increases variability, across all target regions. Furthermore, offline behavioural performance on tasks relevant to the stimulated region/network was also observed to be a factor related to the neurobiological response to TBS (e.g. Annak et al. 2019; Hermiller et al. 2019), suggesting that the effects of TBS are also dependent on external conditions. Task-related neurobiological effects, in the absence of stimulation, are also a critical consideration (Gann et al. 2021a). To elaborate, TBS is mechanistically modelled on oscillatory activity (i.e. theta-gamma coupling) that is a critical mechanism underlying cognitive, most notably memory-related, processes in humans (Lisman and Idiart 1995; Lisman and Jensen 2013; Tamura et al. 2017; Vivekananda et al. 2021). The neurobiological underpinnings of targeted cognitive/behavioural activities are, therefore, a potentially imperative consideration for future research applying TBS to modulate neurobiological or performance-based outcomes. Indeed, using a concurrent TBS-fMRI methodology, Hermiller et al. (2020) report that the effects of TMS are rhythm (TBS vs. beta), network (hippocampal vs. motor), and cognitively (memory/recall) specific. Further work in this area is crucial.

Moving forward, from an experimental perspective, a combination of resting-state and task-based outcomes in future studies would be most informative. Future research should also systematically investigate and report on participant and methodological contributors known to influence TBS outcomes. It is not enough, however, for these factors to be addressed at the level of individual studies. An open-source and shared data repository, combined with a set of established and agreed upon guidelines for basic experimental protocols using TBS, perhaps even with a platform for peer-review of methodology and analysis plans prior to studies being conducted, would address critical methodological limitations in the present literature, and will also contribute to alleviating another large hurdle frequently observed in the literature, i.e., small sample sizes, by allowing for meta-analytic review. Similar programs have been implemented for electrophysiological outcomes of TMS such as MEPs (Corp et al. 2020), and also TMS evoked potentials as measured by electroencephalography (Belardinelli et al. 2019). This could be taken a step further, striving for consistency in TBS protocols between these outcome-focused repositories will, in future, allow for greater understanding of the neurobiological effects of these stimulation protocols beyond compartmentalised outcome measures.

### Summary and conclusion

The present review has summarised and synthesized the literature investigating fMRI-based outcomes of TBS to the cortex of healthy human adults. Although outcomes are often variable, some consistencies emerged when examining resting-state outcomes of TBS applied at motor, parietal, temporal, occipital and cerebellar regions. Specifically, cTBS appeared to induce inhibitory effects, most consistently by way of reduced rsFC, while iTBS produced the opposite (i.e., facilitatory) effect, increasing rsFC. Results of TBS delivered to the PFC were more variable. This was apparent irrespective of task, state, or specific location. Factors contributing to this increased variability observed in response to prefrontal stimulation, however, are unclear.

There are numerous methodological inconsistencies across studies, as well as individual factors known to affect the response to NIBS, which must be addressed. Future research should aim to thoroughly and systematically investigate the role of individual factors such as age, biological sex, anatomical structure, and genetic/epigenetic factors on TBS outcomes. Methodologically, more consistency is needed across studies to facilitate greater integration of findings and data sharing initiatives. Where possible, resting-state and task-based outcomes should both be measured within studies, as well as structural and functional imaging protocols. Care should be taken when determining appropriate comparisons and control targets. Such efforts will be of great benefit to the enhancement of the field, improving outcomes for experimental research studies, and therefore, translation into clinical programs.

In conclusion, factors such as task and state cannot be overlooked when developing future studies and should be considered when determining the most appropriate TBS protocol. More research investigating the effects of known, as well as identifying currently unknown, sources of variability regarding the response to TBS is critical.

## Data Availability

Not applicable.

## Abbreviations

APB: Abductor pollicis brevis
BOLD: Blood oxygen level dependent
CBF: Cerebral blood flow
cTBS: Continuous theta burst stimulation
DAN: Dorsal attention network
DC: Degree centrality
DLPFC: Dorsolateral prefrontal cortex
DMN: Default mode network
FC: Functional connectivity
fMRI: Functional magnetic resonance imaging
HCN: Hippocampal-cortical network
HGal: Anterolateral Heschl’s gyrus
IFG: Inferior frontal gyrus
IPL: Inferior parietal lobe
iTBS: Intermittent theta burst stimulation
LTD: Long-term depression
LTP: Long-term potentiation
M1: Primary motor cortex
MEP: Motor evoked potential
MRI: Magnetic resonance imaging
MRS: Magnetic resonance spectroscopy
NIBS: Non-invasive brain stimulation
OFC: Orbitofrontal cortex
PFC: Prefrontal cortex
PMv: Ventral premotor cortex
PRISMA: Preferred reporting items for systematic reviews and meta-analysis
pSTS: Posterior superior temporal sulcus
rsFC: Resting-state functional connectivity
rTMS: Repetitive transcranial magnetic stimulation
S1: Primary somatosensory cortex
S2: Secondary somatosensory cortex
SMA: Supplementary motor area
TBS: Theta burst stimulation
TMS: Transcranial magnetic stimulation
TPJ: Temporopatietal junction
V1: Primary visual cortex (medial striate area)
V2: Visual area 2
V3: Visual area 3
